# CTCF: an R/bioconductor data package of human and mouse CTCF binding sites

**DOI:** 10.1093/bioadv/vbac097

**Published:** 2022-12-16

**Authors:** Mikhail G Dozmorov, Wancen Mu, Eric S Davis, Stuart Lee, Timothy J Triche, Douglas H Phanstiel, Michael I Love

**Affiliations:** Department of Biostatistics, Virginia Commonwealth University, Richmond, VA 23298, USA; Department of Pathology, Virginia Commonwealth University, Richmond, VA 23284, USA; Department of Biostatistics, University of North Carolina-Chapel Hill, Chapel Hill, NC 27514, USA; Curriculum in Bioinformatics and Computational Biology, University of North Carolina at Chapel Hill, Chapel Hill, NC 27599, USA; Department of Econometrics and Business Statistics, Monash University, Clayton, NC 3168, Australia; Molecular Medicine Division, Walter and Eliza Hall Institute, Parkville, VIC 3052, Australia; Center for Epigenetics, Van Andel Research Institute, Grand Rapids, MI 49503, USA; Department of Pediatrics, College of Human Medicine, Michigan State University, East Lansing, MI 48824, USA; Department of Translational Genomics, Keck School of Medicine, University of Southern California, Los Angeles, CA 90033, USA; Curriculum in Bioinformatics and Computational Biology, University of North Carolina at Chapel Hill, Chapel Hill, NC 27599, USA; Thurston Arthritis Research Center, University of North Carolina at Chapel Hill, Chapel Hill, NC 27599, USA; Department of Cell Biology and Physiology, University of North Carolina at Chapel Hill, Chapel Hill, NC 27599, USA; Lineberger Comprehensive Cancer Center, University of North Carolina at Chapel Hill, Chapel Hill, NC 27599, USA; Curriculum in Genetics and Molecular Biology, University of North Carolina at Chapel Hill, Chapel Hill, NC 27599, USA; Department of Biostatistics, University of North Carolina-Chapel Hill, Chapel Hill, NC 27514, USA; Department of Genetics, University of North Carolina at Chapel Hill, Chapel Hill, NC 27514, USA

## Abstract

**Summary:**

CTCF (CCCTC-binding factor) is an 11-zinc-finger DNA binding protein which regulates much of the eukaryotic genome’s 3D structure and function. The diversity of CTCF binding motifs has led to a fragmented landscape of CTCF binding data. We collected position weight matrices of CTCF binding motifs and defined strand-oriented CTCF binding sites in the human and mouse genomes, including the recent Telomere to Telomere and mm39 assemblies. We included selected experimentally determined and predicted CTCF binding sites, such as CTCF-bound cis-regulatory elements from SCREEN ENCODE. We recommend filtering strategies for CTCF binding motifs and demonstrate that liftOver is a viable alternative to convert CTCF coordinates between assemblies. Our comprehensive data resource and usage recommendations can serve to harmonize and strengthen the reproducibility of genomic studies utilizing CTCF binding data.

**Availability and implementation:**

https://bioconductor.org/packages/CTCF. Companion website: https://dozmorovlab.github.io/CTCF/; Code to reproduce the analyses: https://github.com/dozmorovlab/CTCF.dev.

**Supplementary information:**

Supplementary data are available at *Bioinformatics Advances* online.

## 1 Introduction

The structural and regulatory organization of the mammalian genome is fundamentally dependent on CTCF (CCCTC-binding factor), a versatile transcription regulator evolutionary conserved from fruit fly to human (Kim *et al.*, 2007; Ohlsson *et al.*, 2001; Phillips and Corces, 2009). It has been found to be involved in a variety of regulatory functions including transcriptional activation, imprinting, X-chromosome activation, cancer and developmental disorders, and chromatin interactions in three dimensions (Ohlsson *et al.*, 2010; Phillips and Corces, 2009). CTCF binds DNA through the combinatorial use of its 11-zinc-finger domains to target sites with remarkable sequence variation (Jolma *et al.*, 2013; Ohlsson *et al.*, 2001). A 15–20 bp CTCF consensus motif from the ChIP-seq data analysis has been defined (Kim *et al.*, 2007), referred to hereafter as M1. This motif interacts with the central zinc fingers (ZFs 3–7) for most CTCF–DNA-binding events. Subsequently, a shorter 9 bp motif (M2) interacting with the C-terminal fingers was discovered that, together with M1, forms 34–35 bp CTCF binding sites that cover 5–10% of the total number of CTCF binding events (Hashimoto *et al.*, 2017; Nakahashi *et al.*, 2013; Schmidt *et al.*, 2012). The complexity of CTCF binding was also noted in the earlier study of motif discovery in conserved noncoding elements by Xie *et al.* (2007), detecting LM3, LM7 and LM23 motifs as parts of CTCF binding. Studies have reported 20 000–50 000 CTCF binding sites within the human and mouse genomes (Kim *et al.*, 2007; Nakahashi *et al.*, 2013).

CTCF binding is generally conserved between different tissues (Kim *et al.*, 2007). We aimed to provide uniformly detected strand-specific CTCF binding sites for human and mouse genomes, generally applicable for any cell type. Given the differences in genome assemblies, we defined CTCF binding sites directly for each assembly, including the Telomere to Telomere (T2T) human genome assembly (Nurk *et al.*, 2022) and the GRCm39/mm39 mouse genome assembly. We demonstrate that coordinate conversion (liftOver) is a viable way to obtain assembly-specific CTCF binding sites. We further demonstrate the need to filter less significant CTCF binding sites and merge overlapping sites. Given the importance of CTCF binding in convergent orientation for forming chromatin loops (Rao *et al.*, 2014), we provide strand-specific CTCF binding sites indicating the directionality of CTCF binding. We also include selected experimental CTCF binding sites and assemble them in the CTCF R/Bioconductor data package.

## 2 Implementation

Position weight matrices (PWMs) (Stormo, 2000) of CTCF sequence motifs from Jaspar 2022 (Castro-Mondragon *et al.*, 2022), HOCOMOCO v11 (Kulakovskiy *et al.*, 2018), Jolma 2013 (Jolma *et al.*, 2013) and SwissRegulon (Pachkov *et al.*, 2013) databases were downloaded from the MEME Motif database (Supplementary Table S1). They were largely similar except for Jolma 2013 and two long (34 and 35 bp) Jaspar PWMs (Supplementary Fig. S1A). The CTCF binding site database (CTCFBSDB) (Ziebarth *et al.*, 2013) includes PWMs for the M1+M2 motifs (Schmidt *et al.*, 2012), the Ren_20 motif (Kim *et al.*, 2007) and the LM2, LM7 and LM23 motifs (Xie *et al.*, 2007) (Supplementary Fig. S1B). The CIS-BP database (Weirauch *et al.*, 2014) lists 83 human-specific and 2 mouse-specific PWMs (Supplementary Fig. S1C and D). These PWMs were used to scan the human and mouse genomes for CTCF binding motifs using the FIMO tool from the MEME suite (Bailey *et al.*, 2009).

**Fig. 1.**
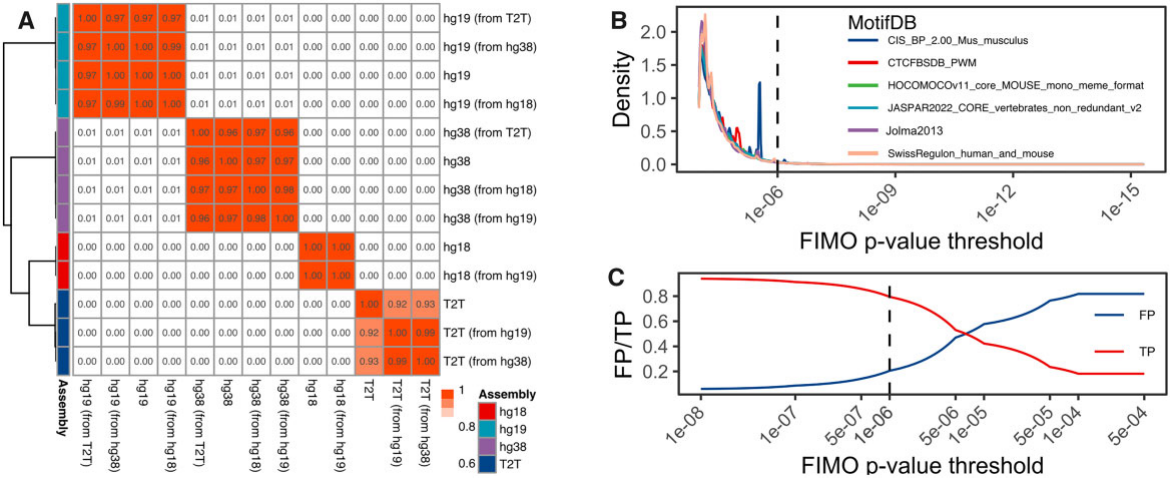
Properties of CTCF motifs detected by FIMO. (**A**) Jaccard overlaps among CTCF binding sites detected in the original and liftOver human genome assemblies. CTCF sites were detected using JASPAR 2022 MA0139.1 PWM. The correlogram was clustered using Euclidean distance and Ward.D clustering. White-red gradient indicate low-to-high Jaccard overlaps. Jaccard values are shown in the corresponding cells. (**B**) Density plot of the number of motifs depending on the FIMO *P*-value threshold. Dashed line—1E−6 *P*-value cutoff. (**C**) The proportion of true/false positive CTCF binding motifs depending on the FIMO *P*-value threshold. ENCODE SCREEN data was used as ground truth

The CTCFBSDB database (Bao *et al.*, 2008) is a dedicated resource of predicted CTCF binding sites for hg18 and mm8 genome assemblies (Supplementary Table S2). As coordinate conversion (liftOver) is a well-established practice, we investigated how CTCF sites detected in one genome assembly compare with those converted from another. Jaccard overlap among CTCF sites aligned or lifted over to hg18, hg19, hg38 and T2T genomes showed that genome assembly was the primary driver of similarity (Fig. 1A). These results were also observed using mm9/mm10/mm39 genome assemblies (Supplementary Fig. S2A), indicating that liftOver is a viable option to obtain CTCF sites in the required genome assembly. Using liftOver, we provide hg19/hg38 and mm9/mm10 versions of predicted CTCF sites from the CTCFBSDB database.

The FIMO tool is one of the oldest and most well-known tools for motif scanning. It uses a dynamic programming algorithm to convert log-odds scores of motif matching into *P*-values (Cuellar-Partida *et al.*, 2012), with the default *P*-value cutoff 1E−4. However, we observed that up to 98% of CTCF sites are detected within the 1E−4 to 1E−6 *P*-value range (Fig. 1B, Supplementary Fig. S2B), suggesting most CTCF sites have a relatively poor sequence match. Using the ENCODE SCREEN V3 database of CTCF-bound cis-regulatory elements (cCREs) (ENCODE Project Consortium *et al.*, 2020) as ground truth (Supplementary Table S2, included in the package), we quantified the proportion of true and false positives depending on the FIMO *P*-value cutoff. We found the 1E−6 threshold the most optimal, providing ∼80% of true positive CTCF motifs (Fig. 1C, Supplementary Fig. S2C). To investigate whether FIMO-detected CTCF sites detected below this threshold (*P*-value > 1E−6, referred hereafter as less significant) may be associated with cell type-specific CTCF binding (Essien *et al.*, 2009), we used 206 cell type-specific experimental CTCF profiles from the UCSC Genome Browser Database (Chen *et al.*, 2012; Lee *et al.*, 2022). We found less significant CTCF sites overlapping on average 7.43 ± 29.24 cell type-specific CTCF sites, in contrast to more significant CTCF sites overlapping on average 120.51 ± 81.19 cell type-specific CTCF sites. Furthermore, only 21.84% of less significant CTCF sites overlapped at least one cell-type-specific CTCF sites, in contrast to 88.80% for more significant CTCF sites (Wilcoxon *P*-value < 2.2E−16). These results suggest that the loss of cell-type-specific information by filtering less significant CTCF sites is minimal; however, they do not exclude the possibility that less significant CTCF sites, in addition to be cell-type specific, may have weaker binding and be missed by conventional peak callers.

Given some databases provide multiple CTCF PWMs, one CTCF site may be detected multiple times resulting in overlapping CTCF sites. Examples include ∼40% overlapping sites in JASPAR2022 data generated using three CTCF matrices, or data from the CTCFBSDB database containing 50–60% overlapping sites, in contrast to 2.5% in data generated using one MA0139.1 matrix. Reducing them (merging overlapping CTCF sites), combined with 1E−6 cutoff filtering, yields the number of CTCF sites comparable to previously reported ones (62 000 on average, Supplementary Table S3). However, regulatory elements with CTCF proteins co-occupying adjacent/overlapping CTCF binding motifs were shown to be functionally and structurally different from those with single CTCF motifs (Pugacheva *et al.*, 2015). We provide non-reduced CTCF data and advise considering the overlap of CTCF sites depending on the study goals.

## 3 Discussion

Our goal was to provide easy programmatic access to CTCF binding data applicable to any cell or tissue type. The CTCF package contains 51 GRanges objects of strand-specific CTCF motifs predicted using 98 PWMs from six databases on human and mouse genome assemblies, including the T2T and mm39 assemblies (Supplementary Table S3). Given the popularity of the JASPAR database, we recommend using predictions made with the MA0139.1 PWM, which will also detect the long M1+M2 sites (Nakahashi *et al.*, 2013), selecting sites detected at 1E−6 *P*-value cutoff and merging overlapping sites. If experimentally detected CTCF sites are of interest, we suggest using CTCF-bound cCREs from the ENCODE SCREEN database. We hope the CTCF package will enable standardization and reproducibility of studies employing CTCF binding data.

## Supplementary Material

vbac097_Supplementary_Data

## Data Availability

The data underlying this article are available via https://bioconductor.org/packages/CTCF. The datasets were derived from sources in the public domain: UCSC, http://genome.ucsc.edu; GitHub, https://github.com/marbl/CHM13; MEME Motif Databases, https://meme-suite.org; CTCFBSDB, https://insulatordb.uthsc.edu.
