# Supplementary material for: CTCF: an R/bioconductor data package of human and mouse CTCF binding sites
Source: Bioinform Adv. 2022 Dec 16;2(1):vbac097. doi: 10.1093/bioadv/vbac097 (PMC9793704; doi:10.1093/bioadv/vbac097)
Supplement: vbac097_Supplementary_Data [file vbac097_supplementary_data.zip › Supplementary_Table_legends.docx]

Supplementary Table Legends

**Supplementary Table S1. CTCF PWM information.** “Motif” - individual motif IDs or the total number of motifs per database; “Length” - motif length of the range of lengths; “URL” - direct links to motif pages. Jaspar, Hocomoco, Jolma 2013 PWMs were downloaded from the MEME database.

**Supplementary Table S2. Predefined CTCF binding data.** “Database” - source of data; “Number” - number of binding sites; “Assembly” - genome assembly; “URL” - direct link to data download.

**Supplementary Table S3. Summary of CTCF binding data provided in the package.** CTCF sites for each genome assembly and PWM combination were detected using FIMO. “ID” - object names formatted as <assembly>.<database name>; “Assembly” - genome assembly, T2T - telomere to telomere (GCA_009914755.4) genome assembly; “All (p-value threshold Xe-Y)” - the total number of CTCF binding sites in the corresponding BED file at the Xe-Y threshold; “Non-overlapping (p-value threshold Xe-Y)” - number of non-overlapping CTCF binding sites (overlapping regions are merged) at the Xe-Y threshold.
