# Supplementary figures and images for: CTCF: an R/bioconductor data package of human and mouse CTCF binding sites
Source: Bioinform Adv. 2022 Dec 16;2(1):vbac097. doi: 10.1093/bioadv/vbac097 (PMC9793704; doi:10.1093/bioadv/vbac097)

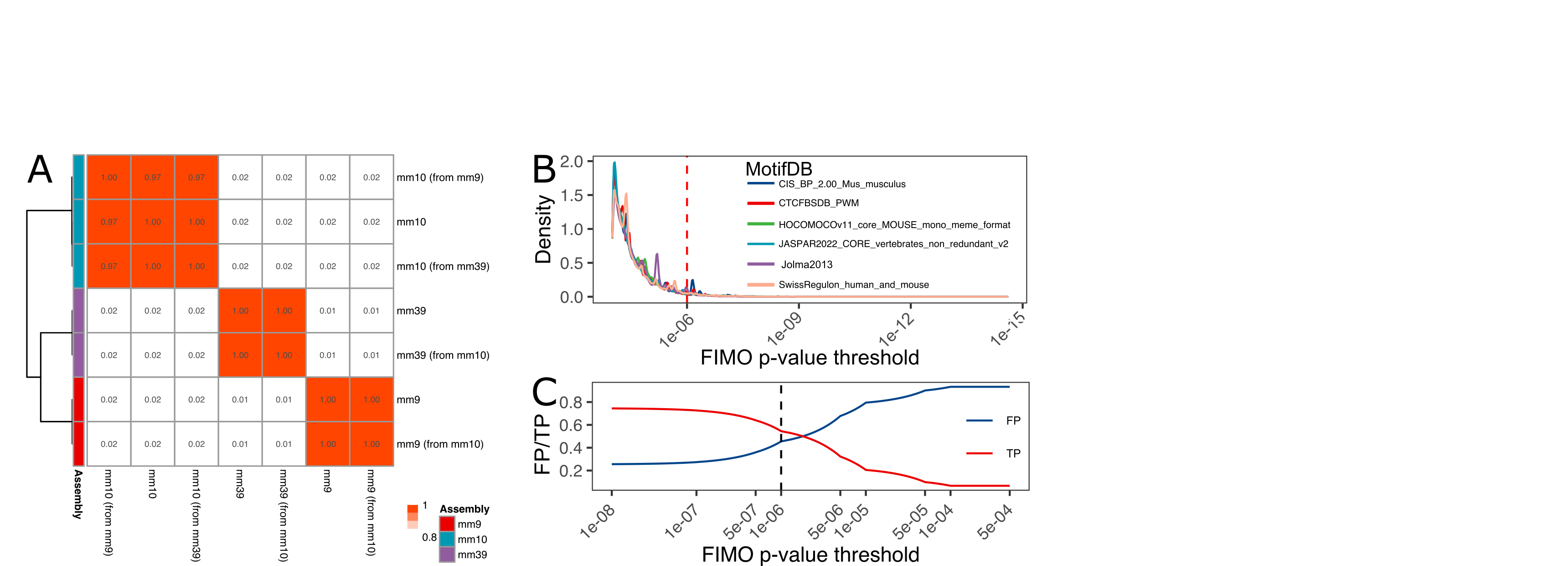

Supplement: vbac097_Supplementary_Data [file vbac097_supplementary_data.zip › Supplementary_Figure_2.png]

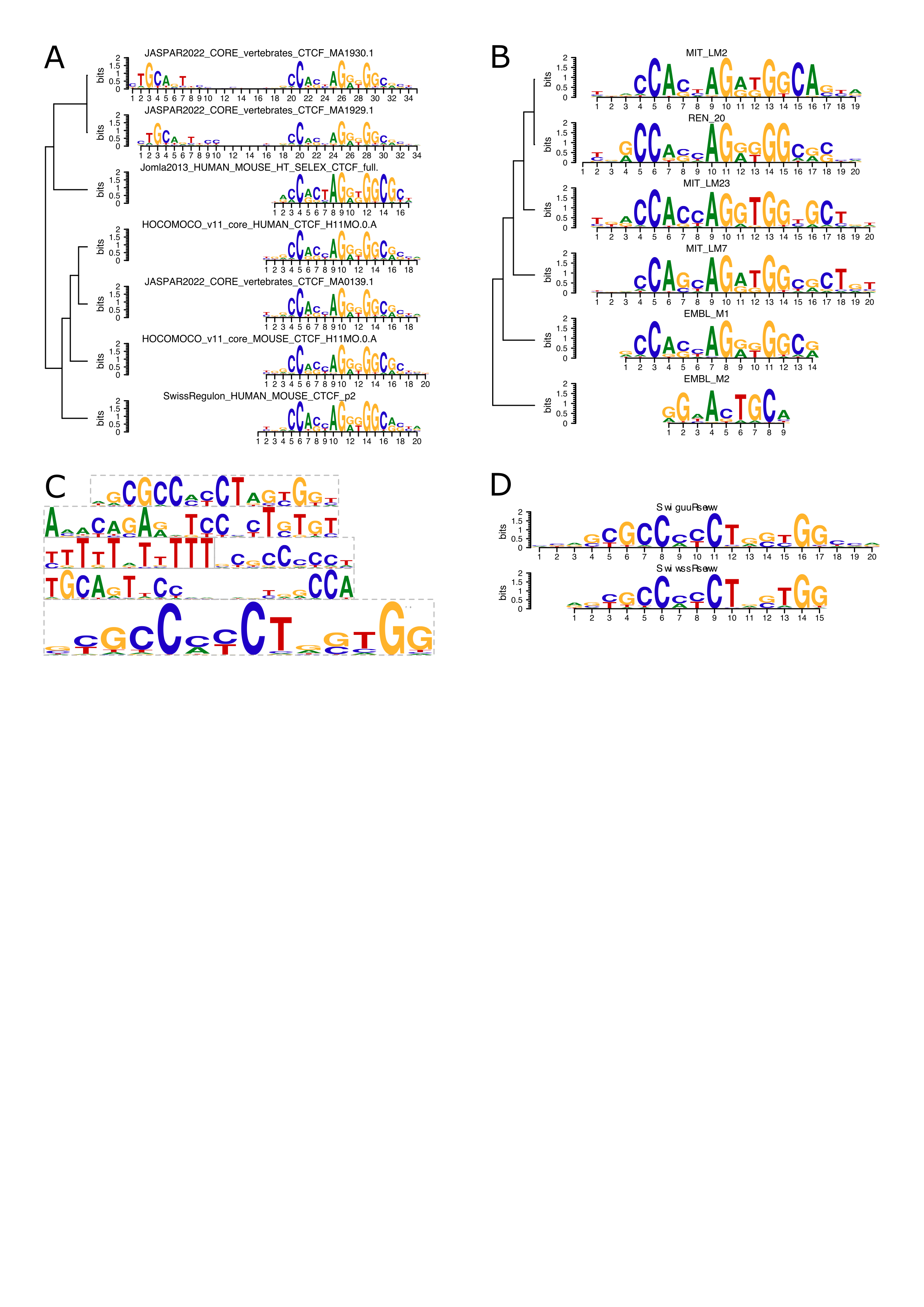

Supplement: vbac097_Supplementary_Data [file vbac097_supplementary_data.zip › Supplementary_Figure_1.png]
